# Comparison of outcomes between trimodal therapy and radical cystectomy in muscle-invasive bladder cancer: a propensity score matching analysis

**DOI:** 10.18632/oncotarget.16576

**Published:** 2017-03-25

**Authors:** Yeon Joo Kim, Sang Jun Byun, Hanjong Ahn, Choung-Soo Kim, Beom-Sik Hong, Sangjun Yoo, Jae-Lyun Lee, Young Seok Kim

**Affiliations:** ^1^ Department of Radiation Oncology, Asan Medical Center, University of Ulsan, College of Medicine, Songpa-Gu, Seoul, Republic of Korea; ^2^ Urology, Asan Medical Center, University of Ulsan, College of Medicine, Songpa-Gu, Seoul, Republic of Korea; ^3^ Oncology, Asan Medical Center, University of Ulsan, College of Medicine, Songpa-Gu, Seoul, Republic of Korea

**Keywords:** muscle-invasive bladder cancer, radical cystectomy, trimodal therapy, radiotherapy, chemotherapy

## Abstract

Although radical cystectomy (RC) is considered as the standard therapy for muscle-invasive bladder cancer (MIBC), trimodal therapy (TMT) combining transurethral resection of the tumor with radiotherapy and chemotherapy is increasingly recommended as an alternative approach for bladder preservation. In the absence of randomized trials, we compared the clinical outcomes between RC and TMT using propensity score matching with 50 patients in the RC arm and 29 patients in the TMT arm. With respective median follow-up periods of 23 and 32 months for the RC and TMT groups, 5-year distant metastasis-free survival (58% vs. 67%), overall survival (56% vs. 57%), and cancer-specific survival (69% *vs*. 63%) rates between the RC and TMT groups, respectively, were similar. However, the 5-year local recurrence-free survival was significantly better in the RC group than in the TMT group (74% *vs*. 35%). Following TMT, acute grade 3 hematological (*n* = 2) and late grade 3 genitourinary (*n* = 1) toxicities were reported. These findings demonstrated that oncological outcomes of TMT were comparable with those of RC, except for poorer local control. Large-scale, randomized trials are warranted to confirm the findings of the present retrospective comparison and to guide toward best treatment options.

## INTRODUCTION

Although radical cystectomy (RC) has been the mainstay treatment for muscle-invasive bladder cancer (MIBC), many patients with several comorbidities are unfit for RC. In addition, median patient age at MIBC diagnosis is about 70 years. As some patients strongly prefer to retain their own bladders, interest in trimodal therapy (TMT), which combines transurethral resection (TUR) of bladder tumor with radiotherapy (RT), and chemotherapy, is increasingly considered as alternative therapy for MIBC. TMT is the most effective bladder-preserving treatment approach for MIBC [1, 2]; however, to date, a prospective randomized trial directly comparing RC and TMT has not been conducted. Several studies showed that overall survival (OS) with TMT was similar to that achieved with RC [3, 4]. Nevertheless, accumulating evidence from several studies indicated that patients receiving TMT tended to be older and had more comorbidities and poorer performance status than those treated with surgery alone, making accurate comparisons impossible. Discrepancies between clinical and pathological staging represent another major hurdle in comparisons between the two treatment options. Clinical staging is more likely to underestimate muscle invasion and can occur in up to 50% of patients [5].

Two previous studies with large cohorts reported similar OS rates between the two treatment strategies after multivariable adjustment to decrease bias [6, 7]. However, a meta-analysis comparing RC and TMT for MIBC recently demonstrated that TMT was associated with a slightly better OS rate [8]. Despite the large number of patients, former studies lacked detailed data on patient and treatment characteristics that could impact survival rates. Additional limitations include missing and heterogeneous data.

The present study was thus conducted to evaluate and compare survival rates and failure patterns between RC and TMT for MIBC, with patient diagnosed, treated, and followed at a single tertiary referral center. To incorporate the significance of treatment effects in the nonrandomized design of our observational study and to decrease the effect of potential confounding factors on outcomes, a propensity score matching analysis was used.

## RESULTS

### Baseline characteristics of patients in RC and TMT groups

Characteristics of 308 patients that underwent RC and 32 patients that received TMT before propensity score matching are listed in Table 1. Patients selected after propensity score matching included 50 and 29 patients in the RC and TMT groups, respectively, as presented in Table 2.

**Table 1:** Patient characteristics according to the treatment group

Characteristics		RC (*n* = 308)	TMT (*n* = 32)	*p*-value
Median age, years (range)		65 (29-82)	77 (40-88)	< 0.01
Gender	Male Female	260 48	25 7	0.38
ASA classifications	1 2 3	62 240 6	5 23 4	< 0.01
Grade	1 2 3	3 29 276	0 2 30	0.82
Multiplicity	Solitary Multiple Unknown	104 203 1	7 25 0	0.31
Clinical T stage	2 3 4	145 142 21	18 13 1	0.49
Hydronephrosis	Present Absent	72 236	1 31	0.01
No. of TURB before RT or surgery	0 1 ≥ 2	11 190 107	0 15 17	0.09
Completeness of TURB	Complete Incomplete Unknown	155 142 11	23 9 0	0.05
Neoadjuvant chemotherapy	Yes No	26 282	8 24	0.01

ASA, American Society of Anesthesiologists; RC, radical cystectomy; RT, radiotherapy; TMT, trimodal therapy; TURB, transurethral resection of bladder tumor

**Table 2:** Patient characteristics according to the treatment after matching for the propensity score

Characteristics		RC (*n* = 50)	TMT (*n* = 29)	*p*-value
Median age, years (range)		72 (48-82)	76 (40-88)	0.67
Gender	Male Female	38 12	23 6	0.67
ASA classifications	1 2 3	8 41 1	5 20 4	0.10
Grade	2 3	1 49	1 28	1.00
Multiplicity	Solitary Multiple	10 40	7 22	0.60
Clinical T stage	2 3 4	33 16 1	16 12 1	0.76
Hydronephrosis	Present Absent	2 18	1 28	1.00
No. of TURB before RT or surgery	0 1 ≥ 2	1 20 29	0 15 14	0.58
Completeness of TURB	Complete Incomplete	35 15	20 9	0.98
Neoadjuvant chemotherapy	Yes No	10 40	7 22	0.60

In the TMT group, eight patients underwent RT alone because of poor performance status (*n* = 6) and/or insufficient renal function (*n* = 2). Thirteen patients (45%) received concurrent cisplatin at a dose of 70 mg/m^2^ every 3 weeks, whereas seven patients underwent neoadjuvant chemotherapy as well. Concurrent and adjuvant chemotherapy was administered in one patient. As neoadjuvant and adjuvant chemotherapy, four cycles of gemcitabine at 1,000 mg/m^2^ combined with cisplatin at 70 mg/m^2^ on day 1 and followed by gemcitabine 1,000 mg/m^2^ on day 8 were administered at 3-week intervals. In both treatment arms, the majority of patients (88% in RC, 97% in TMT) did not receive adjuvant treatment. Median RT dose was 60 Gy (range, 50-60 Gy); 16 patients (55%) received 60 Gy, whereas RT with 50 Gy was administered to only one patient. Intensity-modulated RT (IMRT) was utilized in 25 out of 29 TMT patients. Following TMT, most patients showed a complete (*n* = 21, 72%) or partial (*n* = 3, 10%) response, whereas four patients (14%) experienced disease progression.

Among a total of 13 patients with bladder-only recurrence in the TMT group, four patients with carcinoma *in situ* (Tis, *n* = 2) and T1 (*n* = 2) underwent salvage cystectomy, whereas eight patients (Ta, *n* = 1; Tis, *n* = 3; T1, *n* = 1; T2, *n* = 3) underwent additional salvage TUR. One patient who was simultaneously diagnosed with metastatic stomach cancer was not treated for bladder recurrence. After salvage cystectomy, an 86-year-old male died because of postoperative ileus and sepsis, whereas 59-year-old male experienced postoperative urine leakage and sepsis, which required 2 months of hospitalized care. Eight patients underwent salvage TUR; of these, five patients, including two T2 patients, achieved complete resection.

### Survival rates in RC and TMT groups before and after propensity score matching

**Figure 1:**
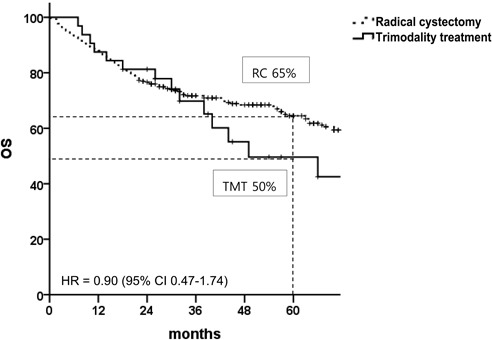
Overall survival in patients with muscle-invasive bladder cancer before propensity score matching HR, hazard ratio; CI, confidence interval; TMT, trimodal therapy; RC, radical cystectomy.

Figure 1 shows OS rates in the unmatched patient population with median follow-up times of 43 months (range, 1-110 months) for the RC group and 31 months (range, 1-107 months) for the TMT group. There was no statistical difference in the 5-year OS between the two arms (RC *vs*. TMT, 65% *vs*. 50%; hazard ratio [HR], 0.90; 95% confidence interval [CI], 0.47-1.74). Among a total of 32 patients in the TMT arm, 5-year local recurrence-free survival (LRFS), distant metastasis-free survival (DMFS), and bladder-intact survival rates were 32%, 66%, and 77%, respectively. Comparison of survival endpoints between the two groups are presented in Table 3. With the exception of LRFS, there were no statistically significant differences in other survival endpoints between the two groups.

**Table 3:** Hazard ratios for survival outcomes according to treatment strategy

Endpoints	5-year survival		HR	95% CI	p-value
	RC (%)	TMT (%)			
Overall survival					
Unadjusted population (univariate)	65	50	0.90	0.47-1.74	0.76
Unadjusted population (multivariate)	-	-	0.87	0.47-1.60	0.65
Matching population	56	57	0.89	0.39-2.03	0.78
Local recurrence-free survival*	74	35	4.18	1.33-13.14	0.01
Distant metastasis-free survival*	58	67	0.79	0.32-1.93	0.60
Cancer-specific survival*	69	63	0.96	0.38-2.47	0.94

Abbreviations: RC, radical cystectomy; RT, radiotherapy; HR, hazard ratio; CI, confidence interval

*only for matched population.

**Figure 2:**
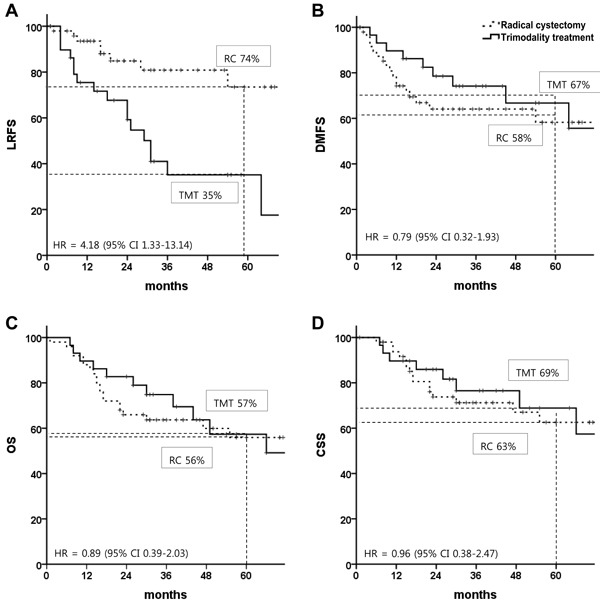
Survival rates in patients with muscle-invasive bladder cancer after propensity score matching: A. local recurrence-free survival, B. distant metastasis-free survival, C. overall survival, and D. cancer-specific survival HR, hazard ratio; CI, confidence interval; TMT, trimodal therapy; RC, radical cystectomy

Figure 2 shows survival curves of the matching populations in both treatment arms. The median follow-up periods of survivors were 23 months (range, 1-107 months) and 32 months (range, 7-93 months) for the RC and TMT groups, respectively. Although the 5-year LRFS rate was significantly better in the RC group than in the TMT group (74% *vs*. 35%; HR, 4.18; 95% CI, 1.33-13.14), the 5-year DMFS 58% vs. 67%; HR, 0.79; 95% CI, 0.32-1.93), OS (56% *vs*. 57%; HR, 0.89; 95% CI, 0.39-2.03), and cancer-specific survival (CSS; 69% *vs*. 63%; HR, 0.96; 95% CI, 0.38-2.47) rates were similar between the two treatment strategies.

Toxicities associated with TMT are shown in Table 4. Most of the acute toxicities were grade 1 or 2. Two cases of acute grade 3 hematological toxicities were resolved with transfusion. A late grade 3 genitourinary toxicity occurred in a 73-year-old female patient who experienced recurrent hematuria 1 year after TMT and was treated with hematoma evacuation and permanent percutaneous nephrostomy insertion.

**Table 4:** Toxicities associated with trimodal therapy (*n* = 29)

Toxicities	Grade 1 (%)	Grade 2 (%)	Grade 3 (%)
Acute GU Upper GI Lower GI Hematologic	14 (48) 5 (17) 4 (14) 13 (45)	7 (24) 3 (10) 6 (21) 6 (21)	0 (0) 0 (0) 0 (0) 2 (7)
Chronic GU GI	4 (14) 0 (0)	3 (10) 0 (0)	1 (3) 0 (0)

GU, genitourinary; GI, gastrointestinal.

## DISCUSSION

Although the 5-year OS rate was not significantly different between the two modalities in the current study, RC showed far better local control than TMT, in line with the findings from previous results. A surgical series reported that the 10-year LRFS ranged from 84% to 97% in lymph node (LN)-negative MIBC patients [9–11], whereas the 5-year LRFS in several Radiation Therapy Oncology Group (RTOG) trials ranged between 39% and 71% [12–14]. Bladder recurrence, the main component of local failure, was observed in 13 out of the 17 patients who experienced local recurrence in the present analysis. A pooled analysis of six RTOG protocols also reported that bladder recurrence was a major characteristic of local failure, with a 5-year bladder recurrence-free survival rate of 57% [3]. Compared with other TMT series, the present study showed poorer bladder control, which we attribute to the following reasons: (1) in contrast the abovementioned clinical trials, most of the patients in the current study were not ideal candidates for bladder preservation by TMT. According to the clinical guidelines, indications for TMT in MIBC include T2 stage, solitary tumor, tumor size < 5 cm, complete TUR of bladder tumor, absence of hydronephrosis, and normal bladder function [15, 16]. However, 26 out of the 29 TMT patients included in the current analysis did not meet these criteria. Furthermore, they were also deemed unfit for RC by urological surgeons. For example, majority of the patients in the current study had multiple tumors (76%) and thus were not ideal candidates for TMT, reflective of a real-life clinical situation; (2) because all patients but one in the current study were treated continuously without restaging TUR, the study cohort could have potentially included a subgroup of non-responders to induction chemoradiotherapy who might have had more residual tumors; (3) the RT dose in the current study appears to be insufficient. As most patients were treated continuously without treatment break for restaging TUR, a 60-Gy radiation dose was given with less concern over tumor repopulation during the treatment.

RT dose escalation is a potentially effective solution to poor bladder control. A phase I trial that evaluated the feasibility of simultaneous integrated tumor boost (to 70 Gy) resulted in the absence of local muscle-invasive recurrence and low rates of late toxicity [17]. Accurate localization of the target should be a prerequisite for dose escalation. In this trial, meticulous bladder filling using a Foley catheter during boost RT and daily cone-beam computed tomography (CT) were implemented; however, some patients were intolerant to the procedure and delineating the residual tumor/tumor bed on cone-beam CT images was difficult in these patients. Recently introduced novel procedures for improved localization of the primary bladder tumor or tumor bed include intramuscular lipiodol injection and implantation of fiducial markers [18, 19]; however, their utility has not yet been verified in large-scale patient populations.

Although there is no consensus on salvage treatment for bladder recurrence, cystectomy is commonly recommended for both non-MIBC and MIBC recurrences. Two of the four patients who received salvage cystectomy in the current study experienced postoperative morbidities, one of which resulted in death. Eswara et al. reported that delayed salvage cystectomy demonstrated acceptable morbidity; however, the median age of their patient cohort was 69.4 years, which was younger than our TMT group (median age, 76 years) [20]. Conversely, salvage TUR showed promising oncological results in present study. These findings suggest that, similar to the initial treatment, TUR and not salvage cystectomy might be considered as first choice for salvage treatment of recurrent bladder cancer following TMT, particularly in elderly patients.

In the current study, no patient experienced recurrence in pelvic LN areas. Routine regional LN irradiation that might have provided a beneficial effect for the prevention of nodal recurrence. The 5-year pelvic nodal recurrence rate has been reported as 13% in the pooled analysis of six RTOG protocols [3]. In contrast, a phase III trial has argued that elective nodal irradiation was unnecessary. Specifically, the investigators compared the oncological outcomes and toxicities between whole-pelvic and bladder-only concurrent chemoradiotherapy regimens and concluded that patients in the bladder-only treatment group experienced less acute toxicity without compromising survival rates [21]. Three-dimensional conformal RT was implemented in that trial; therefore, the exact role of pelvic LN irradiation should be reexamined in the IMRT era. As shown in the IMRT series, acute toxicity was as infrequently reported with whole-pelvic RT as with small-field RT [22].

This study has limitations because of its retrospective design, which is inherently susceptible to selection bias, and the small number of patients. However, all patients were diagnosed and treated in a single tertiary medical center, which minimized heterogeneity in diagnosis and treatment. The data were analyzed using propensity score matching to decrease the impact of treatment selection bias and potential confounding factors that are often encountered in observational studies. On the basis of our previous attempts, a large phase III trial comparing RC and TMT might not be easily implemented [23]. In the absence of a randomized trial, we believe that the current study provided additional information for both patients and their physicians.

In conclusion, using propensity score matching analysis, we showed that the 5-year DMFS, OS, and CSS rates of TMT were comparable to those of RC, with acceptable toxicity profiles despite poorer local control. Additional efforts such as intramuscular lipiodol injection for better delineation of the residual tumor/tumor bed will be helpful to adopt dose-escalated RT, which in turn might decrease bladder recurrence following TMT. Large-scale, randomized trials are warranted to confirm the findings of present comparison.

## MATERIALS AND METHODS

Between 2007 and 2014, 337 and 36 patients underwent RC and TMT, respectively. Patients with histologically-confirmed, MIBC with no evidence of LN involvement or distant metastases were included in the analysis. In the RC group, 29 patients including those with LN metastasis (*n* = 16), non-urothelial carcinoma histology (*n* = 7), non-MIBC (*n* = 5), and double primary colon cancer (*n* = 1) were excluded. In the TMT group, two patients who refused to complete TMT, one patient histologically diagnosed with non-urothelial carcinoma, and one who did not undergo TUR were excluded. Consequently, clinical data from 308 patients in the RC arm and 32 patients in the TMT arm were reviewed and included for propensity score matching. This study was approved by the local institutional review board, and informed consent was waived.

Initial routine workup consisted of cystoscopy, urine cytology, complete blood count, chemistry profile, CT urography, and chest X-ray or chest CT. Surgical procedures for RC and pelvic LN dissection were previously described by Kwon et al. [24]. The extent of LN dissection was determined by the attending surgeon: standard dissection included distal common iliac, external iliac, internal iliac, obturator, and perivesical LNs, whereas extended dissection included additional LNs at the level of proximal common iliac artery, distal aorta, and vena cava. Urinary diversions, including ileal conduit diversions and orthotopic bladder substitutions, were performed after RC and bilateral pelvic lymphadenectomy.

TMT consisted of maximal TUR, RT, and chemotherapy. The RT dose, 46 Gy with a daily fraction size of 2 Gy, was applied to the bladder and elective pelvic nodal areas. In general, initial clinical target volume (CTV) encompassed the pelvic LN area below the common iliac vessels. Planning target volume (PTV) was generated by expanding the margins around the CTV by 7-10 mm. Following whole-pelvic RT, a boost dose of 14 Gy in seven fractions was delivered to the gross residual tumor or to the tumor bed in patients who achieved complete TUR before RT. At the time of CT-based boost planning, a Foley catheter was inserted into each patient, and normal saline was instilled into the bladder as much as was comfortably tolerable. Boost PTV was defined by expanding the residual tumor/tumor bed margins by 10-15 mm. Both three-dimensional conformal RT and IMRT techniques were used. Daily cone-beam CT image guidance was utilized in patients who treated with IMRT and all boost treatments.

For chemotherapy in patients receiving TMT, cisplatin at a dose of 70 mg/m^2^ was administered concurrently with RT every 3 weeks. Neoadjuvant and adjuvant chemotherapy consisted of four cycles of gemcitabine at 1,000 mg/m^2^ with cisplatin at 70 mg/m^2^ on day 1, and gemcitabine 1,000 mg/m^2^ on day 8 at 3-week intervals. Following treatment completion, patients were followed up every 3 months for the first year, every 6 months for the next 4 years, and annually thereafter. Each follow-up appointment consisted of history taking, physical examination, blood tests, urine culture, and cytology. Cystoscopy, chest X-ray, and CT urography were performed every 6 months for the first year and annually thereafter.

Local recurrence was defined as regional LN recurrence at or below the common iliac bifurcation in the RC group and as in-field recurrence in the TMT group. A malignant tumor in the ureter or urethra was considered a second primary tumor and not a recurrence. The RTOG radiation toxicity grading system was used to assess acute and late toxicities in the TMT group.

The primary endpoint was 5-year OS, and secondary endpoints were 5-year LRFS, DMFS, CSS, and toxicities. Survival was calculated from the date of the surgery in the RC arm and from the end of RT in the TMT arm. All data in this study were summarized as means with standard deviations for continuous variables and frequencies or percentages for categorical variables. Comparisons of categorical variables between the RC and TMT groups were conducted using the chi-square test or Fisher's exact test, and Student's *t*-test was used to compare continuous variables. For propensity score estimation, a logistic regression model based on the following factors was used: age, American Society of Anesthesiologist classification representing performance status and comorbidities, pathological grade, tumor multiplicity, clinical tumor stage, presence of hydronephrosis, number of TURs before the main treatment, extent of TUR, and neoadjuvant chemotherapy. Model calibration procedures were also performed and the discriminating ability of the propensity score was confirmed. A one-to-two matching of propensity scores was conducted using a proprietary Statistical Analysis System (SAS) algorithm (SAS Institute, Cary, NC, USA) that selected the best matching pair in the RC arm for each TMT patient, according to the absolute difference between the propensity scores of the RC and TMT cases. Greedy matching was applied and the control selected for a specific case was the one closest to that case in terms of distance; the maximum allowed distance for matching was set to 0.07. For the primary outcome variable, the 5-year OS rate, we conducted univariate analysis (unadjusted model), as well as multivariate analysis, using the Cox proportional hazards model with the full dataset and a stratified Cox model with the matched pair data. Same analyzes were conducted for the secondary end points such as 5-year LRFS, DMFS, and CSS; however, only the propensity score match results are presented in the Results section. The Kaplan-Meier analysis was also conducted for both treatment groups. All statistical analyzes were performed using SAS^®^ version 9.4.
